# Comparative analysis of university lectures, textbooks, and online video lectures: preferences, engagement, and impact on academic performance among medical students of Nowshera Medical College

**DOI:** 10.1186/s12909-026-09921-1

**Published:** 2026-07-14

**Authors:** Abdul Rafey Ishfaq, Abdullah Afridi, Zarak Saeed, Usman Ullah Khan, Waqas Mohyuddin

**Affiliations:** 1 Nowshera Medical College, Nowshera, Pakistan; 2 Department of Community Medicine, Nowshera Medical College, Nowshera, Pakistan

**Keywords:** Medical education, Learning modalities, Online video lectures, Textbooks, University lectures, Cross-sectional Study

## Abstract

**Background:**

Medical students today rely on a combination of traditional and digital learning modalities, such as university lectures (UL), textbooks (TB), and online video lectures (OVL). While the use of OVL is growing worldwide and in Pakistan, little is known about medical students’ preferences, engagement, and perceptions across these modalities. This study aimed to explore learning behaviours, preferred modalities, and their association with academic performance.

**Methods:**

A cross-sectional survey was carried out at Nowshera Medical College, Pakistan, with the overall study period spanning from July 2024 to August 2025. We surveyed 355 undergraduate students from all academic years through stratified random sampling. The questionnaire asked about students’ demographics, preferred learning modality, frequency of use, and perceived effectiveness. It also assessed student perceptions regarding UL and mandatory attendance policies. Data were analysed using descriptive statistics, chi-square tests, ANOVA, correlation, and regression analyses.

**Results:**

OVL were the most preferred learning modality (71.7%), followed by TB (26.9%) and UL (1.4%). The majority of students used YouTube (84.5%) but shifted towards specialized platforms as they entered clinical years. OVL were perceived as the most engaging and effective modality, TB as most helpful for exam confidence and long-term recall, while UL were consistently ranked lowest. Frequency of usage mirrored these views: 92.1% reported often or always using OVL, 88.8% for TB, and just 37.5% for UL. Preferred modality was not significantly linked to exam scores, but students who combined different modalities showed slightly better outcomes. Perceptions of UL and mandatory attendance were generally negative, declining across academic years, though female students rated UL more positively than males.

**Conclusions:**

OVL have become the main source of learning, while TB continue to provide structured guidance. UL, however, received the lowest ratings. Students who used a combination of different learning resources tended to have slightly higher exam scores compared with those who relied on only one. These findings highlight a clear need to integrate digital resources with textbooks, and for UL to be redesigned so that learning is both engaging and effective. Finally, teaching methods should be aligned with students’ actual learning habits in order to optimize outcomes.

**Supplementary Information:**

The online version contains supplementary material available at https://doi.org/10.1186/s12909-026-09921-1.

## Introduction

### Background

In Pakistan, the five-year MBBS program begins with the highly competitive entrance exam (MDCAT). While private colleges are often expensive, public institutions are more affordable. Across all institutions, the Pakistan Medical and Dental Council (PMDC) has a strict 75% minimum attendance policy for annual exam eligibility.

Students today use a variety of learning approaches, ranging from traditional university lectures (UL) and textbooks (TB) to digital platforms such as online video lectures (OVL). Each modality offers its own advantages. UL provide structured guidance and direct interaction with teachers, TB serve as a dependable source of reference material, and OVL platforms (e.g., YouTube, Osmosis, Marrow, PrepLadder) allow students to learn at their own pace and enhance learning by providing visually engaging content [1, 2].

Students typically combine these modalities depending on their personal learning styles, time availability, and perceived effectiveness. Studies from around the world suggest that blended learning models can improve knowledge retention, increase student satisfaction, and enhance academic outcomes [3–5]. In Pakistan, OVL usage is on the rise, with many students finding them more effective for exam preparation and improving grades than UL [6]. However, there still remains a limited understanding of how these modalities interact with each other, highlighting the need to explore their real-world engagement to develop strategies that can maximize learning outcomes.

### Problem / gap

Despite widespread use of multiple learning modalities, evidence on students’ preferences, engagement, and perceptions remains limited, particularly in the context of Pakistani medical colleges. Most existing studies have examined all three modalities separately [7–10], with only a few systematically comparing all three within the same group of students. Moreover, UL have not been rigorously evaluated in terms of student perceptions or educational effectiveness, despite longstanding concerns [11, 12]. In addition, how students shift their reliance on different learning approaches over the course of their studies is not well understood. Finally, we still know little about which platforms (e.g., YouTube, Osmosis, Marrow, and PrepLadder) students prefer, leaving gaps in our understanding of which resources they use the most and how their usage varies among different student groups. Without this, educators and policymakers may struggle to develop teaching strategies that align with students actual learning behaviours, ultimately leading to reduced engagement and weaker academic outcomes.

### Rationale / importance

Understanding student engagement with different learning modalities is crucial for shaping teaching strategies and curricula in ways that truly support their learning. As medical education is shifting towards digital learning, it is important to clarify how traditional methods and modern tools each contribute to learning so when blended models are used, they remain effective and acceptable to students. Comparing the use of learning modalities with academic performance can also provide valuable insight into how students prepare for exams and can identify ways to improve academic outcomes.

Student views on UL and mandatory attendance can help explain the low engagement and guide strategies for making UL more appealing. Similarly, knowing which OVL students prefer can guide decisions on which resources should be formally included in curricula and which to recommend for independent use. With so many students relying on OVL, it becomes crucial to ensure that all students have reliable internet access and institution support to make most of these resources.

These findings carry significant implications for policy, guiding improvements at both institutional and national levels to make medical education more aligned with student learning styles and preferences. They also add to the global debates on the declining role of UL and the rise of OVL, situating Pakistani medical colleges in an international context.

### Objectives of the study

This study aimed to take a comprehensive look at medical students’ learning behaviours and perceptions.

1. To determine students’ preferences for different learning modalities and factors influencing these preferences.
2. To investigate the frequency of engagement with University Lectures (UL), Textbooks (TB), and Online Video Lectures (OVL).
3. To compare the impact of different learning modalities on academic performance.
4. To assess student perceptions of University Lectures and Class Attendance.

The findings are intended to guide educators and policymakers in developing teaching strategies that truly reflect how students learn and help to enhance educational outcomes.

## Methods

### Study design

A cross-sectional survey [13] was carried out at Nowshera Medical College, Pakistan, with the overall study period spanning from July 2024 to August 2025. Data collection from all academic years occurred on a single day. The study was conducted in accordance with the STROBE guidelines [14]. The primary objective was to explore medical students’ preferred learning modalities and the factors influencing their choices. Due to our academic responsibilities, the study was conducted in phases, which included development of questionnaire, data collection, and data entry.

### Setting and participants

Nowshera Medical College is a public-sector institution offering a five-year MBBS program with approximately 120 students per class and a total enrolment of about 600. The college follows a PMDC (Pakistan Medical and Dental Council) mandated integrated modular curriculum where traditional university lectures are neither recorded nor available online. The study included all undergraduate students from first through final year.

We used stratified random sampling, treating academic year and gender as separate strata. Using institutional roll numbers, we randomly selected 40 male and 40 female students per class. To ensure proper representation, selected students who were absent were contacted on the same day. However, due to low overall attendance in the third year (*n* = 58) on the day of data collection, all present third-year students were invited to participate.

Exclusion criteria included (i) minimal engagement (“rarely” or “never”) in at least two of the three learning modalities, (ii) previous exam scores below 55 and (iii) leaving > 10% of the questionnaire blank. Students scoring below 55% were excluded to control for non-academic factors (e.g., chronic absenteeism or personal crises) that cause severe underperformance, ensuring our analysis focused accurately on the normative study habits of actively engaged, passing students.

Using Cochran’s formula for a finite population (*N* ≈ 600) with a 95% confidence level and a 5% margin of error, the minimum required sample was 234 students [15]. To allow for a 10% non response rate, we targeted 260. Ultimately, 378 students completed the questionnaire. After applying exclusion criteria, 23 responses were removed, leaving 355 valid questionnaires for analysis, providing sufficient statistical power for year-wise comparisons.

### Data collection

The research team designed the questionnaire with expert review from the Head of Community Medicine. Questionnaire items were developed through an extensive literature review to ensure relevance to the local educational context. The draft was reviewed by the Head of Department to assess content validity, clarity, and clinical relevance. A pilot test with a small group of students led to the removal of redundant items and further improvements in clarity. The final version of the questionnaire (Supplementary_File_1_Final_Questionnaire.pdf.docx) contained four sections: (1) introduction and demographics, (2) preferences for learning modalities, (3) impact on academic performance, and (4) perceptions of university lectures and attendance.

Data collection took place in classrooms using paper-based questionnaires during a pre-approved 30-minute window between scheduled lectures. During this time, the research team explained the purpose of the study, distributed questionnaires, and remained available to answer any questions. Participation was entirely voluntary, and anonymity and confidentiality were assured. Students were explicitly informed they could return blank questionnaires if they did not wish to participate.

### Variables and measures

Section 1 gathered demographic and background data such as gender, year of study, residence, device access, extracurricular involvement, sleep patterns, and study hours.

Section 2 explored students’ preferences for different learning modalities (University Lectures, Textbooks, Online Video Lectures) using eight newly developed 5-point Likert scale items per modality, measuring frequency of use, impact on understanding complex concepts, clinical knowledge, memorization, engagement, flexibility, satisfaction, and likelihood to recommend. Internal consistency was acceptable: University Lectures (α = 0.855), Textbooks (α = 0.744), and Online Video Lectures (α = 0.651).

Section 3 examined the perceived impact of these modalities on academic performance, including self-reported academic percentages from their main final annual professional examinations (which were not verified against official college records to maintain anonymity), exam preparation practices, confidence, and long-term outcomes.

Section 4 consisted of two newly developed scales based on literature review themes: a six-item University Lecture perception scale and a four-item Attendance perception scale, both using 5-point Likert responses, with higher scores reflecting more favourable perceptions.

Psychometric evaluation of the questionnaire was limited to internal consistency testing. The scales demonstrated acceptable reliability, with Cronbach’s alpha values of 0.875 for the University Lecture Perception Scale and 0.726 for the Attendance Scale after removal of one item. However, advanced validation methods such as exploratory and confirmatory factor analysis were not performed.

### Data analysis

Data were analysed using IBM SPSS Statistics for Windows, Version 27.0 [16]. Descriptive statistics, including frequencies, percentages, means, and standard deviations, were used to summarize participant characteristics and questionnaire responses, while Cronbach’s alpha evaluated scale reliability [17]. Before analysis, Likert items that were wrongly coded were reversed in SPSS.

Chi-square tests were used for categorical variables, while independent-samples t-tests and one-way ANOVA were used to compare means. When the assumptions for these tests were not met, non-parametric tests like Mann–Whitney U, Kruskal–Wallis H, and Friedman tests (with post-hoc Wilcoxon signed-rank tests) were used. Spearman’s correlations explored relationships between continuous variables.

Binary logistic regression was used to identify factors associated with learning method preference (OVL vs. non-OVL), as few students preferred university lectures. Ordinal logistic regression examined factors associated with self-reported exam scores. Both models adjusted for gender, year of study, study hours, exam preparation time, attendance, and recent exam scores. Effect sizes were reported alongside p-values, missing data were handled through predefined exclusion criteria without imputation, and statistical significance was set at *p* < 0.05.

### Ethical considerations

Ethical approval for this study was granted by Ethical Review Board (ERB) of Nowshera Medical College (Reference: 21/ERB/NMC). The research was conducted in line with the institutional ethical guidelines and the principles of Declaration of Helsinki [18]. All participants were briefed on the study’s purpose, methods and their rights, such as right to withdraw at any point. Written consent was obtained by signing the first page of the questionnaire. No identifying details were gathered, ensuring that both confidentiality and participant anonymity were fully protected.

## Results

### Participant characteristics

A total of 378 medical students responded, 23 were excluded based on predefined criteria, leaving 355 for analysis. Gender distribution was nearly equal, and participants were roughly evenly distributed across the academic years. Most students lived in hostels (70.4%) and studied between 7-12 h daily during annual exam preparation days (Table 1).

**Table 1:** Participant characteristics of medical students (*n* = 355)

Variable	Category	Frequency (*n*)	Percentage (%)
Gender	**Male**	173	49
	**Female**	182	51
Academic Year	**1st Year**	78	22
	**2nd Year**	74	21
	**3rd Year**	55	16
	**4th Year**	74	21
	**Final Year**	74	21
Average Daily Sleep	**< ** **4 h**	15	4
	**4 - 6 h**	150	42
	**6 - 8 h**	170	48
	**> ** **8 h **	20	6
Weekly Study Hours	**< ** **6 h**	128	36
	**6 - 12 h**	128	36
	**13 - 18 h**	62	18
	**19 - 24 h**	15	4
	**25 - 30 h**	15	4
	**> 30 h**	7	2
Exam Preparation Hours	**< 4 h**	13	5
	**4 - 6 h**	23	8
	**7 - 9 h**	105	38
	**10 - 12 h**	84	30
	**> ** **12 h**	52	19

Percentages may not total 100 due to rounding. “Exam Preparation Hours” refers to average daily hours studied in the 1–2 months before exams

### Objective 1: preferences for learning modalities and factors affecting it

OVL was the most preferred learning modality (71.7%), followed by TB (26.9%) and UL (1.4%) among a total of 355 students (Fig. 1). Chi-square goodness-of-fit indicated a significant deviation from equal preference (χ² (2) = 267.90, *p* < 0.001).

**Fig. 1:**
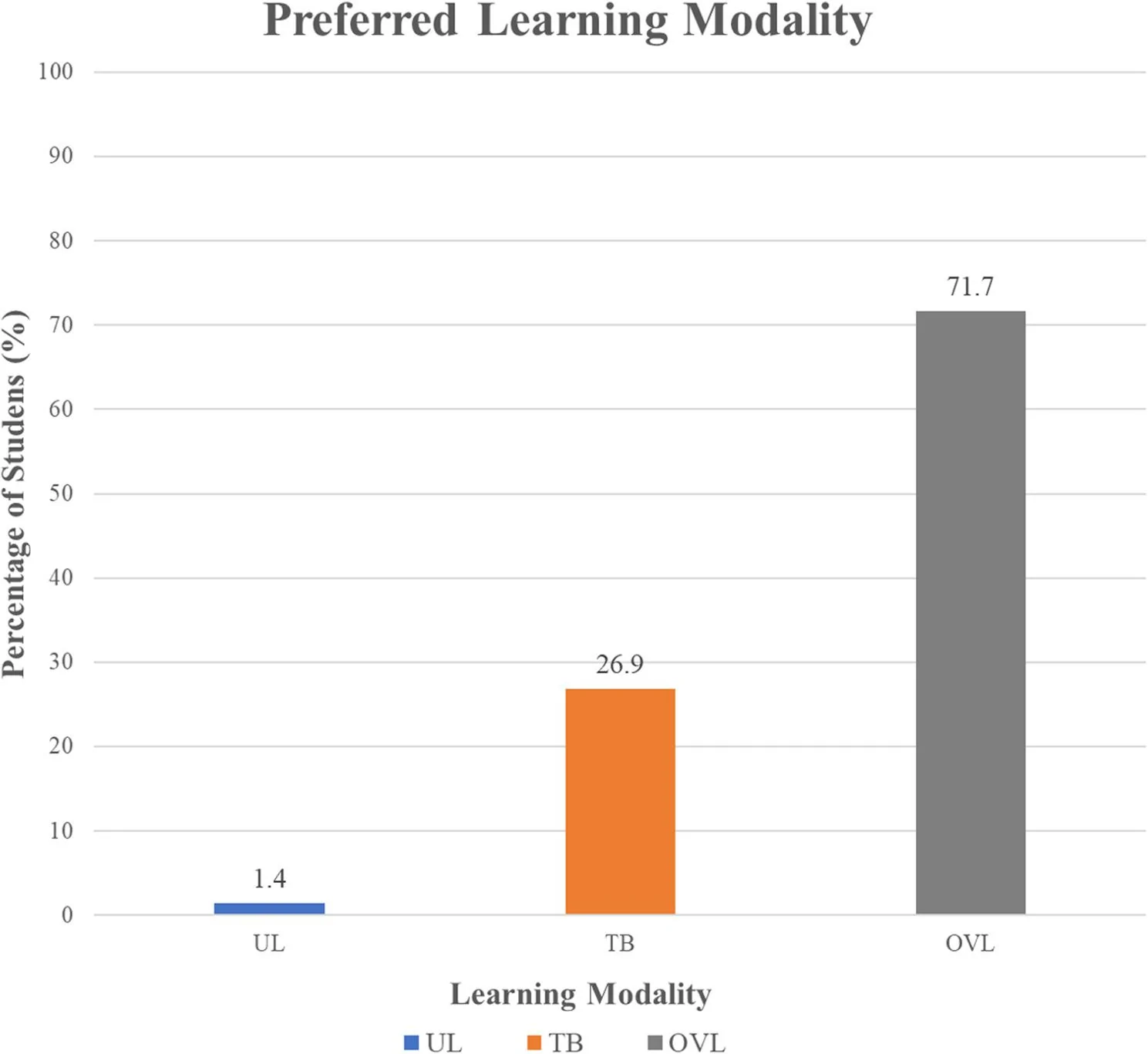
Preferred learning modality among medical students (*n* = 355). UL = University Lectures; TB = Textbooks; OVL = Online Video Lectures

Students reported clear differences in engagement across modalities. OVL were found to be most engaging, with 81% of students rating them highly. TB were rated highly engaging by 49%, while only 7% rated UL as highly engaging. The majority (64%) reported low engagement with UL (Supplementary Table 1).

OVL were rated the most effective for understanding clinical knowledge, with 83.4% of students assigning high effectiveness, compared with 72.1% for TB and 38% for UL (Supplementary Table 2). For topics involving heavy memorization, TB was most preferred (51.9%), followed by OVL (47.9%), while preference for UL was negligible (0.3%).

The analysis focused on the six most frequently used platforms (YouTube, Prepladder, Boards & Beyond, Sketchy, Kaplan, and Osmosis), as other platforms were used by very few students. Among them, YouTube was the most frequently used (84.5%), followed by other platforms (Table 2). Chi-square tests showed that year of study was significantly associated with the use of all six platforms (all ps < 0.001).

**Table 2:** Usage of specific online video lecture platforms across academic years

Platform	1st Year (%)	2nd Year (%)	3rd Year (%)	4th Year (%)	Final Year (%)	Total (%)
YouTube	96.2	91.9	78.2	79.7	77.5	84.5
Prepladder	0.0	0.0	47.3	59.5	75.7	35.5
BNB	0.0	6.8	16.4	67.6	83.8	35.5
Sketchy	1.3	0.0	49.1	64.4	39.2	29.3
Kaplan	0.0	6.8	20.0	35.1	35.1	19.2
Osmosis	2.6	8.1	9.1	31.1	41.9	18.9

Values represent the percentage of students in each academic year who reported using a specific platform (1st Year: *n* = 78; 2nd Year: *n* = 74; 3rd Year: *n* = 55; 4th Year: *n* = 74; Final Year: *n* = 74). Total (%) indicates overall usage across all years

YouTube was the most popular platform across all academic years, but especially among pre-clinical students. In contrast, platforms like Prepladder, BNB, Kaplan, Sketchy, and Osmosis were minimally used by pre-clinical students but their popularity increased sharply from 3rd year onward. These patterns indicate that clinical year students rely more heavily on specialized platforms (Table 2).

Gender differences also showed a clear pattern. Females demonstrated a significantly greater use of YouTube (91.8% vs. 78.2%), Sketchy (34.1% vs. 24.4%) and Osmosis (24.7% vs. 12.7%) than males. In contrast, no significant gender differences were noted for Prepladder, BNB, or Kaplan, as usage rates were similar between genders.

Across the academic years, TB preference declined from 35.9% in first year to 23.0% in final year, with minor fluctuations in intermediate years. Meanwhile, OVL preference increased from 64.1% in first year to 74.3% in second year and stayed stable afterward. Chi-square tests for trend indicated neither change was statistically significant (TB: *p* = 0.143; OVL: *p* = 0.308).

Preferred learning modality was not meaningfully associated with gender, academic year, weekly study hours, or extracurricular participation (all *p* > 0.1; V = 0.034–0.134). Across all subgroups, OVL remained dominant, TB was second, and UL was rarely selected (Fig. 2). First-year students were excluded from analyses involving exam scores and exam preparation study hours.

**Fig. 2:**
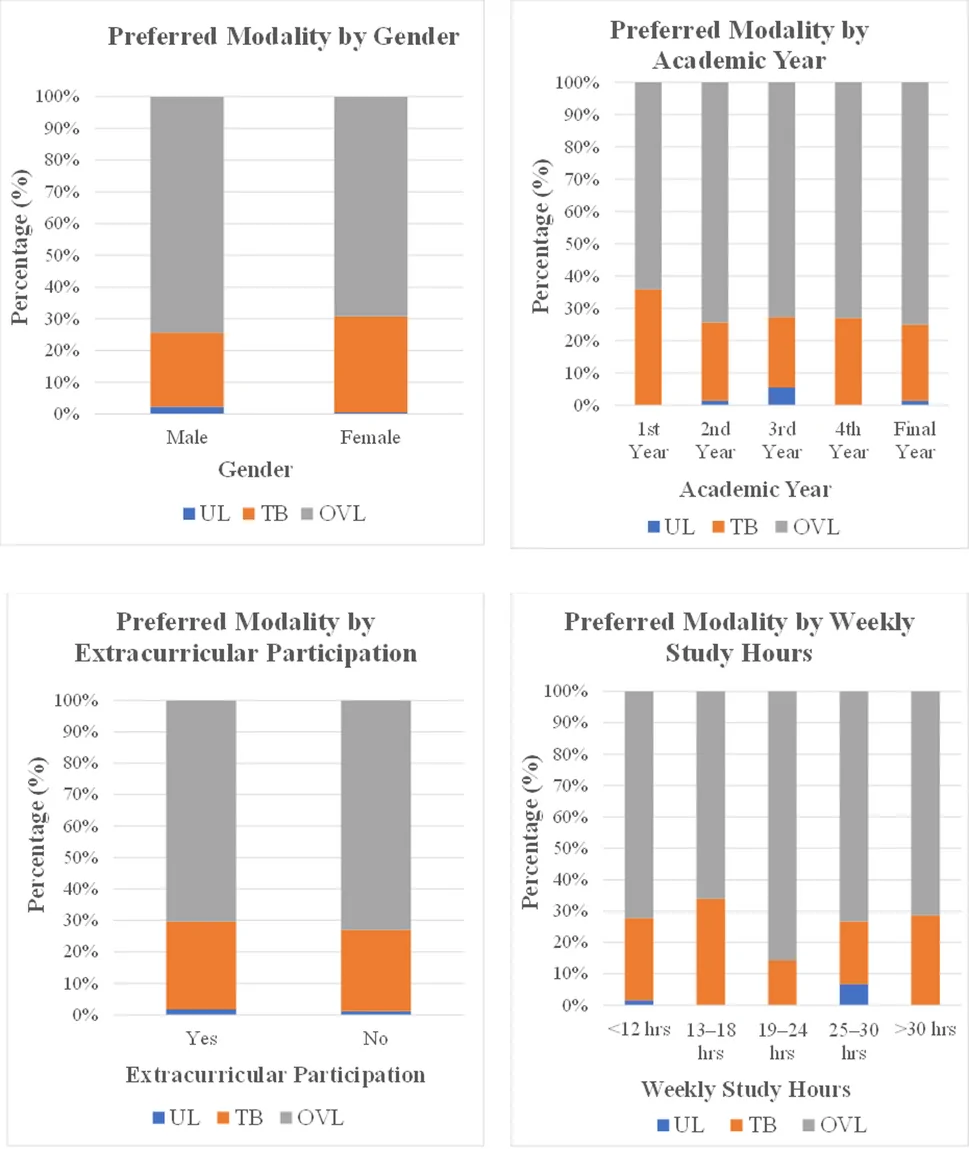
Distribution of preferred learning modality across student characteristics. Characteristics include gender, academic year, extracurricular participation, and weekly study hours. Percentages are shown for each category. UL = University Lectures; TB = Textbooks; OVL = Online Video Lectures

The extent to which classmates’ and seniors’ recommendations influenced learning modality choices differed significantly across response categories (Supplementary Table 3). Most students reported being strongly (40.8%) or moderately influenced (31.5%), with smaller proportions choosing slight (9.3%), or no influence (6.2%).

Students were most likely to recommend OVL (95%), followed by TB (89%) and UL (30%) (Supplementary Table 4). A Friedman test showed recommendation likelihood differed significantly across modalities, χ²(2, *N* = 352) = 430.69, *p* < 0.001, with OVL ranked highest (2.56), followed by TB (2.23) and UL (1.21).

None of the factors—gender, year of study, study hours, attendance, or latest exam score—significantly predicted preference for OVL (χ²(6) = 6.02, *p* = 0.422, Nagelkerke R² = 0.031). For TB, male sex significantly predicted preference (OR = 1.95, *p* = 0.027), while other covariates were non-significant (model χ² (6) = 8.68, *p* = 0.193; Nagelkerke R² = 0.046).

### Objective 2: frequency of engagement (UL, TB, OVL)

Descriptive statistics (Table 3, Fig. 3) showed that UL were the least used, with 62.5% of students (*n* = 221) reporting *never* or *rarely* using them for learning. TB were used more frequently, with 88.8% (*n* = 314) indicating *often* or *always*. OVL ranked highest, with 92.1% (*n* = 327) reporting *often* or *always* using this modality.

**Table 3:** Frequency of use of learning modalities (*n* = 354)

Learning Modality	Mean (M)	SD	Never (%)	Rarely (%)	Sometimes (%)	Often (%)	Always (%)
University Lectures (UL)	2.32	1.05	23.2	39.3	23.4	10.7	3.4
Textbooks (TB)	4.45	0.74	0	1.7	9.6	31.3	57.5
Online Video Lectures (OVL)	4.58	0.69	0.3	1.1	6.5	24.5	67.6

1 = Never, 5 = Always. Friedman and post-hoc Wilcoxon tests indicate OVL and TB were used more frequently than UL. OVL vs. TB difference was not significant

*SD* Standard Deviation

**Fig. 3:**
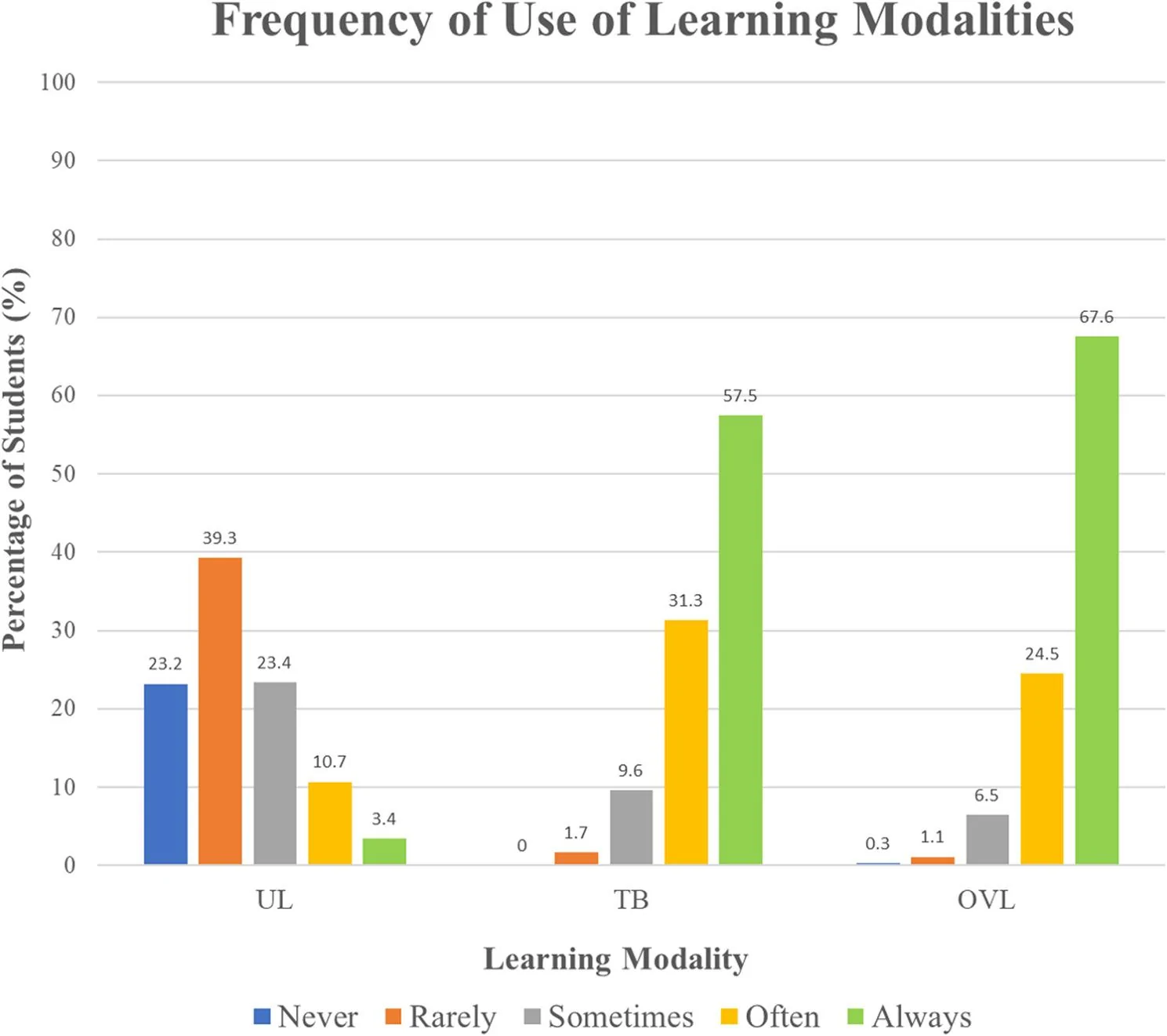
Frequency of use of learning modalities (*n* = 354). OVL was used most frequently, followed by TB and UL. UL = University Lectures; TB = Textbooks; OVL = Online Video Lectures

Friedman test confirmed significant differences across modalities, χ² (2, *N* = 354) = 477.34, *p* < 0.001, with Kendall’s W = 0.674, indicating a large effect size. Both TB and OVL were used significantly more than UL, whereas the difference between TB and OVL was small and not statistically significant (Z = − 2.401, *r* = 0.128).

Frequency of use differed significantly across academic years for all three modalities. First-year students relied most on UL, significantly more than 4th- and final-year students (H = 15.85, *p* = 0.003). TB use was highest among 1st-year students, lower in 3rd- and final-year students (H = 28.38, *p* < 0.001). OVL were used least by first-year students, increased sharply in second year, and remained consistently high in later years (H = 12.24, *p* = 0.016).

Spearman correlations revealed weak positive associations: UL use correlated positively with attendance (ρ = 0.149, *p* = 0.005), TB with weekly study hours (ρ = 0.119, *p* = 0.025), and OVL with exam preparation study hours (*ρ* = 0.193, *p* = 0.001).

### Objective 3: impact on exam performance

Analyses included students with prior exam experience (2nd year and above). Among 273 students, most scored 71–75% (31.1%) and 76**–**80% (25.3%). For exam preparation, 50.7% preferred OVL, 47.4% preferred TB, and 1.9% preferred UL.

Descriptive ratings (Table 4) showed highest confidence for TB, followed by OVL, and lowest for UL; frequency of reliance was greatest for OVL and lowest for UL; perceived long-term impact was positive for TB and OVL and neutral for UL.

**Table 4:** Student confidence, reliance, and perceived long-term impact of learning modalities

Domain	University Lectures (UL)	Textbooks (TB)	Online Video Lectures (OVL)
Confidence (M ± SD)	3.33 ± 1.44	4.24 ± 0.88	3.74 ± 1.23
Frequency of Reliance (M ± SD)	2.49 ± 1.18	4.03 ± 1.17	4.47 ± 0.84
Long-term Impact (M ± SD)	2.95 ± 1.08	4.18 ± 0.88	4.37 ± 0.83

Confidence = students’ self-reported belief in performing well on exams after studying from each modality; Frequency of reliance for exams = how often students used each modality for exam preparation; Long-term impact = perception of each modality’s influence on sustained learning and overall academic performance

*M* Mean, *SD* Standard Deviation

Preferred learning modality showed no associations with latest exam scores (Table 5).

**Table 5:** Latest exam scores by preferred learning modality

Preferred Modality	*N*	Median Exam Score	Mean Rank
University Lectures (UL)	5	4 (71–75%)	119.10
Textbook (TB)	66	4 (71–75%)	136.52
Online Video Lectures (OVL)	200	4 (71–75%)	136.25

Median Exam Score shows the middle score category for each group (coded 1–6). Mean Rank values are taken from the Kruskal–Wallis H test. The test indicated no significant difference in exam performance across modalities (H (2) = 0.252, *p* = 0.881). The exam score is coded as follow): 1 = 56–60%, 2 = 61–65%, 3 = 66–70%, 4 = 71–75%, 5 = 76–80%, 6 = > 80%

Chi-square analysis indicated no significant association between exam performance group (low vs. high scorers) and preferred learning modality, χ²(2, *N* = 271) = 0.05, *p* = 0.973, with a negligible effect size (Cramer’s V = 0.014).

Two hundred ninety-nine students (84.7%) agreed or strongly agreed that using a combination of study modalities is more effective, while 54 students (15.3%) were neutral or disagreed. Belief in combining modalities showed a weak positive correlation with exam scores, ρ (271) = 0.170, *p* = 0.005. However, there were no significant differences in scores across the TB/UL, TB/OVL, or Equal-preference groups, χ² (2) = 0.054, *p* = 0.974.

Ordinal logistic regression further revealed that gender and academic year are significant predictors of exam performance, with male students and those in the third and fourth years less likely to achieve higher score categories. Other factors like preferred learning modality, combination of modalities, exam preparation study hours, and attendance were not significant predictors. The model explained a modest proportion of the variance in exam performance (Nagelkerke R² = 0.121).

### Objective 4: perceptions of university lectures

Overall, students’ perception of UL was below the neutral midpoint (M = 2.57, SD = 0.83) with excellent internal consistency (α = 0.875). Detailed item descriptives are in Table 6.

**Table 6:** Descriptive statistics for university lecture perception items

Item	Mean	SD
Teaching methods are effective	2.70	1.09
Lectures are engaging	2.42	1.01
Content is clear and understandable	2.70	1.05
Comfortable participating/asking questions	2.61	1.10
Lectures help prepare for exams	2.47	1.02
Lectures important for overall academic success	2.52	1.10

Scores on 5-point Likert scale (1 = strongly disagree, 5 = strongly agree)

A one-sample t-test confirmed that overall perceptions were significantly below neutral (t (354) = − 9.76, *p* < 0.001, d = − 0.52), representing a moderate effect. Female students reported higher perception scores than males (Mean Rank = 192.19 vs. 163.08), a statistically significant difference, U = 13,161, *p* = 0.007. Perceptions did not vary significantly across academic years, preferred learning modality or attendance (all *p* > 0.05). Spearman’s correlation with exam performance was weak and non-significant (ρ = − 0.091, *p* = 0.132).

### Objective 5: perceptions of attendance

Students’ perception of attendance was below the neutral midpoint (M = 2.48, SD ≈ 0.85) with acceptable reliability (α = 0.726). Detailed item-level means are in Table 7. Q3 (“Attendance trend”) was excluded to improve internal consistency but is reported descriptively.

**Table 7:** Descriptive statistics for attendance perception items

Item	*N*	Mean	SD
Attendance would be same if not compulsory	355	2.32	1.20
Attendance trend over time	277	3.24	1.09
Attendance impact on exam performance	354	2.61	1.20
Missing lectures affects grades	355	2.04	1.02
Attendance is important for overall academic success	355	2.21	1.12

N varies for some items due to missing responses. Specifically, Attendance Trend had a lower response rate (*N* = 277) because it did not apply to first-year students

A one-sample t-test confirmed the mean was significantly below neutral, t(354) = − 15.73, *p* < 0.001, d = − 0.84, representing a large effect. Although female students reported slightly higher scores than males, this difference was not significant (U = 14,836.5, *p* = 0.346). Perceptions of attendance varied significantly across academic years (Kruskal–Wallis H = 28.36, *p* < 0.001). Post-hoc tests showed that first-year students expressed more positive attitudes than students in all other years (all adj. ps < 0.05), among which no significant differences were noted. Complementing this, Spearman’s correlation indicated a small but significant negative relationship between academic year and attendance perceptions (ρ = –0.20, *p* < 0.001), showing that attitudes toward attendance declined as students progressed through medical school. Finally, Spearman’s correlation with exam performance was very weak and non-significant (*ρ* = − 0.078, *p* = 0.201).

## Discussion

### Introduction

In this study, we explored medical students’ preferences, usage patterns, and perceptions of the three main learning modalities—University Lectures (UL), Textbooks (TB) and Online Video Lectures (OVL)—and their relationship with examination performance. OVL emerged as the most preferred and frequently used modality, whereas TB remained useful for providing structured information. UL were consistently rated lowest in terms of engagement, clarity, and exam relevance. Although reliance on a single learning modality did not significantly affect examination outcomes, students who combined multiple modalities performed slightly better, highlighting the advantages of a multimodal approach. Compulsory attendance was generally seen in a negative light, with students doubting its actual educational value. In short, these findings point to a clear transition in medical education, with students moving away from conventional to the more flexible and digitally-oriented learning methods.

### Comparison with previous literature

Our study revealed that OVL was the most preferred learning modality (71.7%), while UL was rarely chosen. Videos from platforms such as YouTube, Osmosis, UWorld, Anki, and SketchyMedical were among the most preferred study modalities [19]. Tackett et al. reported that 87.9% of students found Osmosis videos helpful, with 57.8% preferring them over lectures [1]. Additionally, 82.3% of medical students used YouTube for learning pathology, which was associated with higher exam scores (≥ 70%; OR 1.96, 95% CI: 1.08–3.57) [20]. These findings highlight the central role OVL now play in exam preparation.

Although TB were not the most frequently used modality, they remained crucial for enhancing exam confidence and long-term retention. This aligns with previous research demonstrating that while modern students increasingly rely on digital supplements, textbooks still provide a critical foundation that is often associated with higher academic achievement, particularly during pre-clinical years [21–23]. Consistent with this trend, our study noted a decrease in TB preference from 35.9% in the first year to 23.0% in the final year, further reflecting the broader shift toward video-based learning in clinical settings.

UL emerged as the least preferred learning modality, earning the lowest ratings in terms of engagement, clarity, and exam relevance. The majority of students had negative views on compulsory attendance, with many doubting its academic contribution. Interestingly, female students had significantly higher perception scores compared to males (Mean Rank = 192.19 vs. 163.08, U = 13,161, *p* = 0.007). This indicates that while the overall ratings for UL were low, female students tended to view UL more favourably than their male counterparts. Consistent with prior studies, in-person lectures were regarded as less effective for exam preparation [24], and attending lectures or accessing recordings did not significantly influence academic outcomes [25]. Nearly 70% of students reported that they found YouTube more engaging than lectures and textbooks [20]. However, studies have also found that attending lectures regularly may boost exam performance [26], especially for high-achieving students who depend heavily on lecture slides for preparation [27]. Overall, these findings suggest that UL may not fully match with learning style of today’s medical students, highlighting the need to make teaching more interactive and student-centered.

We found that most students favoured multimodal learning, as about 85% used more than one type of modality. Similar findings were reported by Al Shmanee et al. (2024), in which 62.6% of students preferred combining physical and digital resources [23]. Other studies have shown that students who combine tools such as podcasts, active learning, and textbooks performed better than those who were relying on a single resource [28]. Together, these results suggest that a blended learning is the most consistently effective strategy.

In summary, OVL dominate learning habits, yet TB continue to provide a stable foundation and UL, if modernized, could regain their educational significance. The evidence also indicates that combining modalities, rather than depending on a single source, is associated with a slight academic advantage.

### Interpretation and implications

OVL emerged as the main learning modality for over two-thirds of students, a pattern consistent across gender, academic year, and study hours. This indicates that OVL is no longer viewed simply as a supporting tool but has become a central component of student learning. For educators, this underlines the need to adapt their methods so they better fit with the way medical students learn in this day and age. Supporting this, a recent study showed that digitizing lectures into short videos with quizzes and feedback not only boosts knowledge, but also improves concentration and decreases anxiety levels when compared with traditional lectures [29]. Global studies after COVID-19 also back up this trend, pointing to the rise of blended learning, new technologies, and greater autonomy in medical education [30].

Although OVL are widely used, they are not without limitations. The quality of publicly available videos can be inconsistent, which can result in inaccuracies or gaps in concepts, as demonstrated in a systematic review of YouTube videos, which revealed wide variability in quality, with most offering limited educational value [31]. In addition, unequal access to stable internet or paid platforms may also put some students at a disadvantage. Many popular OVL are created for global audiences rather than specific local curricula, potentially reducing their usefulness for exam preparation.

All of this suggests that OVL should complement, rather than replace, structured teaching. In this context, TB—though less frequently used—still plays an important role in strengthening exam confidence and supporting long-term retention. Therefore, a blended model that combines OVL with TB may offer the most balanced and effective way to learn.

Our analysis did not reveal a strong link between preferred learning modality and exam performance. However, male students and those in higher years of study tended to score lower, which suggests that factors other than learning preference may influence exam performance. Students who reported studying from multiple modalities tended to have slightly higher exam scores, demonstrating a small positive association. Still, causality cannot be established because of the study’s cross-sectional design. It remains unclear whether mixing resources actually drives better grades, or if highly motivated, top-achieving students are just more likely to use multiple tools. While the link is there, further research is needed to understand the direct educational impact.

Collectively, these results suggest that promoting multimodal learning could help students in performing better on exams. It is important to put these quantitative findings into perspective. Even though links like study hours or changing attendance views were statistically significant, their actual effect sizes were very small, meaning their real-world educational impact might be minor. Because medical school success depends on a complex mix of factors, educators should be careful not to overstate the influence of any single variable. Universities can play a role in this by integrating videos, lecture slides and active learning into their curricula, ultimately creating a more engaging and balanced educational experience.

The consistently low ratings of UL, together with students’ negative perceptions of mandatory attendance, raises important questions about how effective traditional teaching really is. Although UL still provide a solid framework for delivering essential content, they still need to be redesigned to be more interactive rather than simply giving passive information.

Students choose learning resources on their own rather than through formal guidance. They try different online lectures themselves and often rely on classmates and seniors to find what works best for exams. This peer-sharing culture helps filter useful content, but it can also lead to a “herd effect,” where popular platforms are preferred over better but lesser-known ones. Since faculty are not involved in guiding or vetting these choices, a clear gap exists between the formal curriculum and students’ independent digital learning habits.

Mandatory attendance policies may frustrate students who prefer other study methods, highlighting the need for rules that better fit modern learning trends. This frustration appears stronger in senior years, where our data showed a clear drop in positive attitudes toward attendance. Likely reasons include heavier clinical duties, pressure from high-stakes exams, and reliance on efficient digital platforms like Prepladder or BNB. For these students, strict lecture attendance can feel like a poor use of limited and valuable study time.

#### Strengths

The study included a large sample of participants from all academic years, with nearly equal number of male and female students, improving the generalizability of results. Multiple learning modalities were explored, which gave a complete overview of student learning preferences. The study showed strong internal consistency (Cronbach’s alpha 0.651–0.875), supporting the reliability of results. Data completeness was high, and analysing subgroups by gender, academic year, study hours, and extracurricular participation added further depth. Taken together, the results reflect current learning patterns and offer practical guidance for curriculum development.

### Limitations

The cross-sectional, single-institution design of the study limits generalizability and prevents causal conclusions. In addition, data was self-reported which raises the possibility of recall and social desirability bias.

There is a possibility of sampling bias. Some subgroups were small (e.g., only 58 third-year students were present), so random sampling could not be applied. Moreover, although selected absent students were contacted later, students who are frequently absent may still be underrepresented. As these students may differ from regular attendees, the findings could be slightly affected.

Furthermore, our strict exclusion criteria (removing students scoring below 55%) likely biased the sample toward higher-performing, engaged individuals which may have reduced our ability to detect meaningful links between learning preferences and exam scores.

Statistically, we ran multiple tests without adjustments (such as a Bonferroni correction), increasing the risk of Type I errors. Additionally, we did not formally evaluate the proportional odds assumption for our ordinal logistic regression model, meaning those predictive estimates remain exploratory. Psychometrically, while the questionnaire showed acceptable reliability, we did not perform formal factor analyses to establish structural validity, and the OVL subscale’s internal consistency was slightly lower than ideal (α = 0.651).

Finally, the study’s broad scope meant we could not explore the deeper psychological reasons behind students’ preferences. We also did not account for clinical ward-based teaching in senior years or specific active learning frameworks like Problem-Based Learning (PBL), which is currently limited at our institution. Despite these constraints, the study provides valuable, practical insights into modern student study habits.

### Recommendations and future directions

Future efforts should focus on making UL more engaging and effective by adopting qualities of online resources that students already prefer. These may include high-yield content, concise explanations, adding interactive activities, and using multimedia tools such as short videos or animations. Findings from a three-step digitization model supports this idea, showing that inclusion of videos, interactivity, and feedback can modernize lectures and improve student performance [29]. While replacing UL entirely with external platforms is not realistic, applying their core qualities such as clarity, conciseness, visual support, engagement, and relevance can offer a way to strengthen lecture-based learning.

Colleges should also consider more flexible, competency-based attendance policies for senior students who balance heavy clinical responsibilities. Approaches like flipped classrooms—where students learn theory online and use class time for case discussions—can better integrate digital learning while maintaining the value of in-person teaching.

Future research should go beyond documenting preferences and explore why students favor online video lectures over traditional methods, along with which features make them more effective. Longitudinal and interventional studies are needed to assess whether blended learning models improve long-term retention, clinical competence, and exam performance compared to traditional lectures, especially within local medical curricula.

## Conclusion

This study emphasizes the importance of modernizing medical education by strengthening UL with features that have made OVL effective and engaging for students. Although many students preferred OVL, TB still remain crucial for providing detailed explanations and serving as a dependable guide for exam preparation. Instead of being replaced by OVL, TB should complement them. A blended approach that includes the redesigned UL, OVL, and consistent TB use creates a balanced and comprehensive learning experience. By adapting teaching methods to current learning habits, medical education can remain relevant and ensure that the students are well prepared for the digital era of medical education.

## Supplementary Information

Supplementary Material 1.

Supplementary Material 2.

## Data Availability

The datasets generated and analysed during this study are not publicly available, as they are stored in both physical and electronic formats (Excel and SPSS) by the authors. Data can be obtained from the corresponding author upon reasonable request.

## Abbreviations

MBBS: Bachelor of Medicine, Bachelor of Surgery
UL: University Lectures
TB: Textbooks
OVL: Online Video Lectures
ANOVA: Analysis of Variance
PMDC: Pakistan Medical and Dental Council
STROBE: Strengthening the Reporting of Observational Studies in Epidemiology
