# Supplementary material for: Comparative analysis of university lectures, textbooks, and online video lectures: preferences, engagement, and impact on academic performance among medical students of Nowshera Medical College
Source: BMC Med Educ. 2026 Jul 14;26:1603. doi: 10.1186/s12909-026-09921-1 (PMC13651357; doi:10.1186/s12909-026-09921-1)
Supplement: Supplementary file 1 [file 12909_2026_9921_MOESM1_ESM.docx]

**Supplementary File 1: Final Questionnaire**

This file contains the final version of the questionnaire that was distributed to participants. Some questions from the initial version were manually removed before distribution because they were considered not relevant to the study objectives. The remaining questions were renumbered sequentially.

**Supplementary Table 1**. Student Engagement Ratings Across Learning Modalities (N = 355).

| **Learning Modality** | **Mean ± SD** | **Low Engagement (1–2)** | **Moderate Engagement (3)** | **High Engagement (4–5)** |
| --- | --- | --- | --- | --- |
| **University Lectures** | 2.16 ± 0.98 | 64% | 28% | 7% |
| **Textbooks** | 3.44 ± 0.93 | 15% | 36% | 49% |
| **Online Video Lectures** | 4.14 ± 0.91 | 6% | 13% | 81% |

***Note:*** *Engagement was assessed on a 5-point Likert scale (1 = Very Low, 5 = Very High). Responses were grouped into three categories:* Low Engagement *(1–2),* Moderate Engagement *(3), and* High Engagement *(4–5). Values are presented as mean ± standard deviation, along with category-wise percentages (N = 355).*

**Supplementary Table 2.**  Student Perceived Effectiveness for Understanding Clinical Knowledge Across Learning Modalities (N = 355).

| **Learning Modality** | **Mean ± SD** | **Low Effectiveness (1–2)** | **Moderate Effectiveness (3)** | **High Effectiveness (4–5)** |
| --- | --- | --- | --- | --- |
| **University Lectures** | 3.07 ± 1.13 | 27.3% | 34.7% | 38% |
| **Textbooks** | 3.86 ± 0.90 | 6.4% | 21.4% | 72.1% |
| **Online Video Lectures** | 4.24 ± 1.01 | 6.4% | 10.1% | 83.4% |

***Note:*** *Effectiveness was rated on a 5-point Likert scale (1 = Very Low, 5 = Very High). For clarity, responses were grouped into three categories:* Low Effectiveness *(1–2),* Moderate Effectiveness *(3), and* High Effectiveness *(4–5). Values are presented as mean ± standard deviation along with category-wise percentages (N = 355).*

**Supplementary Table 3.** Influence of classmates’ and seniors’ recommendations on choice of learning modality (N = 355).

| **Level of influence** | **Number of Students (n)** | **Percentage of Students (%)** |
| --- | --- | --- |
| **Do not influence** | 22 | 6.2 |
| **Slightly influence** | 33 | 9.3 |
| **Moderately influence** | 112 | 31.5 |
| **Strongly influence** | 145 | 40.8 |
| **Very strongly influence** | 43 | 12.1 |

**Note:** Percentages reflect the proportion of students selecting each level.

**Supplementary Table 4.** Likelihood of recommending each Learning Modality (N = 355).

| **Learning Modality** | **Unlikely** | **Neutral** | **Likely** |
| --- | --- | --- | --- |
| **University Lectures** | 38.5% | 31.7% | 29.7% |
| **Textbooks** | 2.5% | 8.8% | 88.7% |
| **Online Video Lectures** | 3.1% | 1.7% | 95.2% |

***Note:*** *Response options were originally measured on a 5-point scale (Very Unlikely, Unlikely, Neutral, Likely, Very Likely). For clarity, categories were combined into three groups:* Unlikely *(Very Unlikely + Unlikely),* Neutral*, and* Likely *(Likely + Very Likely). Percentages reflect the proportion of students selecting each category. Percentages may not sum to 100% due to rounding.*
