# Supplementary material for: Comparative analysis of university lectures, textbooks, and online video lectures: preferences, engagement, and impact on academic performance among medical students of Nowshera Medical College
Source: BMC Med Educ. 2026 Jul 14;26:1603. doi: 10.1186/s12909-026-09921-1 (PMC13651357; doi:10.1186/s12909-026-09921-1)
Supplement: Supplementary file 2 [file 12909_2026_9921_MOESM2_ESM.docx]

**Introduction to the Questionnaire**

**Title:**

**“Comparative Analysis of University Lectures, Textbooks, and Online Video Lectures: Preferences, Engagement, and Impact on Academic Performance Among Medical Students of Nowshera Medical College”**

Dear Participant,

We are students of 4th Year MBBS Nowshera Medical College conducting a research study. The purpose of our research is to investigate the preferences of medical students for different learning modalities (University Lectures, Textbooks, and Online Video Lectures), understand factors associated with them and the impact of these learning modalities on academic performances. Your responses will allow us to analyse these learning modalities in great detail and depth.

**Instructions:**

- The questionnaire consists of several questions divided into four sections.
- Please answer each question carefully based on your experience and perspectives.
- Choose one option unless it is specified that you may choose multiple options.
- Whenever a response box is provided, please enter your answer in the box.

**Confidentiality:**

Your involvement in this study will remain anonymous. We ensure that your response will remain confidential. The data will be stored securely and will solely be used for the purpose of this study.

**Voluntary Participation:**

Participation in this study is entirely voluntary. You may choose to stop participating at any time you want.

**Note:**

All your study habits, preferences and opinions are valid. There is no right or wrong answer.

**Contact Information:**

If you have any questions, please contact Abdul Rafey at [abdulrafey214@gmail.com](mailto:abdulrafey214@gmail.com)

**Consent to Participate in Research:**

By continuing with this questionnaire, you have agreed to participate in this study.

Thank you for your significant contribution to this study!

Signature: _______________

**SECTION 1: INTRODUCTION and DEMOGRAPHIC**

**1.** What is your gender?

☐ Male	☐ Female

**2.** **What is your current academic year?**

☐ 1st Year MBBS	☐ 2nd Year MBBS	☐ 3rd Year MBBS	☐ 4th Year MBBS	☐ Final Year MBBS

**3.** What is your residence as a student?

☐ Hostelite	☐ Day Scholar

**4.** Do you have reliable access to a Tablet/laptop for studying?

☐ Yes	☐ No

**5.** Are you involved in any extracurricular activities?

☐ Yes	☐ No

If yes, how many hours per week do you spend on these activities?

☐ Less than 4 hours	☐ Between 4-7 hours	☐ Between 7-10 hours	☐ More than 10 hours

**6.** How many hours of sleep do you get on an average night?

☐ Less than 4 hours	☐ 4-6 hours	☐ 6-8 hours	☐ More than 8 hours

**7.** On a weekly basis, how many hours per week do you study **(excluding classes and wards)**

☐ Less than 6 hours per week	☐ 6-12 hours per week	☐ 13-18 hours per week ☐ 19-24 hours per week	☐ 25-30 hours per week ☐ More than 30 hours per week

**8.** During the one to two months preparation period (preps) before the annual exams, how many hours per day do you typically spend studying**? (Do not attempt this question if you are in 1st year)**

☐ Less than 4 hours per day	☐ 4-6 hours per day	☐ 7-9 hours per day	☐ 10-12 hours per day	☐ More than 12 hours per day

**Section 2: Preferences of Learning Modalities**

**1.** What is your most preferred learning modality for studying?

☐ University Lectures	☐Textbooks	☐ Online Video Lectures (Utilizing online video lectures from various sources)

How frequently do you use the following study modalities for your learning?

| Study Resource | Never | Rarely | Sometimes | Often | Always |
| --- | --- | --- | --- | --- | --- |
| University Lectures | ☐ | ☐ | ☐ | ☐ | ☐ |
| Textbooks | ☐ | ☐ | ☐ | ☐ | ☐ |
| Online Video Lectures | ☐ | ☐ | ☐ | ☐ | ☐ |

**2. If you use online video lectures for studying, which platforms or sources do you use? (Select all that apply)**

☐ Youtube	☐ Prepladder	☐ Boards and Beyond(BNB)	☐ Osmosis	☐ Kaplan	☐ Lecturio	☐ Dams	☐ Sketchy	☐ Others (please specify):

__________________________________________

☐ I do not use online video lectures.

**3.** Which learning modality do you prefer the most for understanding complex concepts?

☐ University lectures	☐ Textbooks	☐ Online Video Lectures

How do you perceive the impact of each learning modality on your understanding of complex medical concepts?

| Learning Modality | Very High | High | Moderate | Low | Very Low |
| --- | --- | --- | --- | --- | --- |
| University Lectures | ☐ | ☐ | ☐ | ☐ | ☐ |
| Textbooks | ☐ | ☐ | ☐ | ☐ | ☐ |
| Online Video lectures | ☐ | ☐ | ☐ | ☐ | ☐ |

**4.** Which learning modality do you prefer the most for understanding clinical knowledge and/or skills?

☐ University lectures	☐ Textbooks	☐ Online Video Lectures

To what extent do you agree that each modality helps you understand clinical knowledge and/or skills?

| Study Modality | Strongly Disagree | Disagree | Neutral | Agree | Strongly Agree |
| --- | --- | --- | --- | --- | --- |
| University Lectures | ☐ | ☐ | ☐ | ☐ | ☐ |
| Textbooks | ☐ | ☐ | ☐ | ☐ | ☐ |
| Online Video Lectures | ☐ | ☐ | ☐ | ☐ | ☐ |

**5.** Which learning modality do you prefer for subjects or topics that involve heavy memorization?

☐ University lectures	☐ Textbooks	☐ Online video lectures

For subjects or topics that involve heavy memorization, how effective is each of the following modalities in helping you perform better?

| Modality | Very Effective | Effective | Neutral | Ineffective | Very Ineffective |
| --- | --- | --- | --- | --- | --- |
| University Lectures | ☐ | ☐ | ☐ | ☐ | ☐ |
| Textbooks | ☐ | ☐ | ☐ | ☐ | ☐ |
| Online Videos | ☐ | ☐ | ☐ | ☐ | ☐ |

**6.** Do you find a combination of different modalities more effective than using just one?

☐ Strongly Agree	☐ Agree	☐ Neutral	☐ Disagree	☐ Strongly Disagree

How often do you combine University Lectures with Textbooks for studying?

☐ Always	☐ Often	☐ Sometimes	☐ Rarely	☐ Never

How often do you combine Online Video Lectures with Textbooks for studying?

☐ Always	☐ Often	☐ Sometimes	☐ Rarely	☐ Never

**7.** How much do the recommendations of your classmates and seniors influence your choice of learning modality?

☐ Very Strongly influence	☐ Strongly Influence	☐ Moderately Influence	☐ Slightly Influence	☐ Do Not Influence

**8.** Which modality do you find most engaging/interesting for learning new material?

☐ University Lectures	☐ Textbooks	☐ Online Video Lectures

How engaging/interesting do you find each of the three learning modalities?

| Study Modality | Not Engaging | Slightly Engaging | Moderately Engaging | Very Engaging | Extremely Engaging |
| --- | --- | --- | --- | --- | --- |
| University Lectures | ☐ | ☐ | ☐ | ☐ | ☐ |
| Textbooks | ☐ | ☐ | ☐ | ☐ | ☐ |
| Online Video Lectures | ☐ | ☐ | ☐ | ☐ | ☐ |

**9.** How flexible is each modality in accommodating your personal learning pace?

| Study Modality | Very Flexible | Flexible | Neutral | Inflexible | Very Inflexible |
| --- | --- | --- | --- | --- | --- |
| University Lectures | ☐ | ☐ | ☐ | ☐ | ☐ |
| Textbooks | ☐ | ☐ | ☐ | ☐ | ☐ |
| Online Video Lectures | ☐ | ☐ | ☐ | ☐ | ☐ |

**10.** Overall, how satisfied are you with each of the following learning modalities?

| Learning Modality | Very Satisfied | Satisfied | Neutral | Unsatisfied | Very Unsatisfied |
| --- | --- | --- | --- | --- | --- |
| University Lectures | ☐ | ☐ | ☐ | ☐ | ☐ |
| Textbooks | ☐ | ☐ | ☐ | ☐ | ☐ |
| Online Video Lectures | ☐ | ☐ | ☐ | ☐ | ☐ |

**11.** How likely are you to recommend each learning modality to a classmate or junior?

| Study Modality | Very Likely | Likely | Neutral | Unlikely | Very Unlikely |
| --- | --- | --- | --- | --- | --- |
| University Lectures | ☐ | ☐ | ☐ | ☐ | ☐ |
| Textbooks | ☐ | ☐ | ☐ | ☐ | ☐ |
| Online Video Lectures | ☐ | ☐ | ☐ | ☐ | ☐ |

**Note**: If you are in first year of MBBS, please ignore this question.

**12.** How does your preference for university lectures, textbooks, and online video lectures change as you progress through your academic years?

| Study Activity | Strongly Disagree | Disagree | | Neutral | Agree | Strongly Agree |
| --- | --- | --- | --- | --- | --- | --- |
| My preference for university lectures has increased | ☐ | ☐ | | ☐ | ☐ | ☐ |
| My preference for textbooks has increased. | ☐ | ☐ | | ☐ | ☐ | ☐ |
| My preference for online video lectures has increased | ☐ | ☐ | | ☐ | ☐ | ☐ |
|  | | |  | | | |

**Section 3: Impact on Academic Performance**

**1.** What percentage did you score in each academic year**? (Skip if you are in first year)**

| Academic Year | Percentage Range |
| --- | --- |
| 1st Year | ☐ **50-55%** ☐ **56-65%** ☐ **66-70%** ☐ **71-75%** ☐ **76-80%** ☐ **More than 80%** |
| 2nd Year | ☐ **50-55%** ☐ **56-65%** ☐ **66-70%** ☐ **71-75%** ☐ **76-80%** ☐ **More than 80%** |
| 3rd Year | ☐ **50-55%** ☐ **56-65%** ☐ **66-70%** ☐ **71-75%** ☐ **76-80%** ☐ **More than 80%** |
| 4th Year | ☐ **50-55%** ☐ **56-65%** ☐ **66-70%** ☐ **71-75%** ☐ **76-80%** ☐ **More than 80%** |

**2.** What is your preferred learning modality when preparing for exams?

☐ University Lectures	☐ Textbooks	☐ Online Video Lectures

How frequently do you rely on each in order to prepare for exams?

| Study Modality | Always | Often | Sometimes | Rarely | Never |
| --- | --- | --- | --- | --- | --- |
| University Lectures | ☐ | ☐ | ☐ | ☐ | ☐ |
| Textbooks | ☐ | ☐ | ☐ | ☐ | ☐ |
| Online Video Lectures | ☐ | ☐ | ☐ | ☐ | ☐ |

**3.** How confident are you in performing well on exams after studying through these learning modalities?

| Learning Modality | Very Confident | Confident | Neutral | Unconfident | Very Unconfident |
| --- | --- | --- | --- | --- | --- |
| Textbooks | ☐ | ☐ | ☐ | ☐ | ☐ |
| Online Video Lectures | ☐ | ☐ | ☐ | ☐ | ☐ |
| University Lectures | ☐ | ☐ | ☐ | ☐ | ☐ |

**4.** How do you rate the impact of University Lectures, Textbooks, and Online Video Lectures on your long-term academic performance?

| Learning Modality | Very Positive | Positive | Neutral | Negative | Very Negative |
| --- | --- | --- | --- | --- | --- |
| University Lectures | ☐ | ☐ | ☐ | ☐ | ☐ |
| Textbooks | ☐ | ☐ | ☐ | ☐ | ☐ |
| Online Video Lectures | ☐ | ☐ | ☐ | ☐ | ☐ |

**Section 4: University Lectures and Attendance**

**(4a) University lectures**

**1.** I find the teaching methods used in university lectures to be effective.

☐ Strongly Agree	☐ Agree	☐ Neutral	☐ Disagree	☐ Strongly Disagree

**2.** University lectures are engaging and hold my interest**.**

☐ Strongly Agree	☐ Agree	☐ Neutral	☐ Disagree	☐ Strongly Disagree

**3.** The content presented in university lectures is clear and understandable.

☐ Strongly Agree	☐ Agree	☐ Neutral	☐ Disagree	☐ Strongly Disagree

**4.** I feel comfortable asking questions and participating during university lectures.

☐ Strongly Agree	☐ Agree	☐ Neutral	☐ Disagree	☐ Strongly Disagree

**5.** University lectures are effective in helping me prepare for exams**.**

☐ Strongly Agree	☐ Agree	☐ Neutral	☐ Disagree	☐ Strongly Disagree

**6.** University lectures are important for my overall academic success.

☐ Strongly Agree	☐ Agree	☐ Neutral	☐ Disagree	☐ Strongly Disagree

**(4b) Attendance**

**1.** What is your overall attendance for this academic year?

☐ 0-20%	☐ 20-40%	☐ 40-60%	☐ 60-80%	☐ 80-100%

**2.** My attendance would be the same if the attendance was not compulsory.

☐ Strongly Agree	☐ Agree	☐ Neutral	☐ Disagree	☐ Strongly Disagree

**3. How has your overall attendance generally changed as you have progressed through your academic years?**

☐ **Increased**	☐ **Decreased**	☐ **Remained the Same**	☐ **No specific pattern**	☐ Not sure

**4.** University attendance has a significant impact on my examination performance.

☐ Strongly Agree	☐ Agree	☐ Neutral	☐ Disagree	☐ Strongly Disagree

**5.** Missing lectures affects my grades negatively.

☐ Strongly Agree	☐ Agree	☐ Neutral	☐ Disagree	☐ Strongly Disagree

**6.** University attendance is important for my overall academic success.

☐ Strongly Agree	☐ Agree	☐ Neutral	☐ Disagree	☐ Strongly Disagree
